# Prognostic evaluation of stage I lung adenocarcinoma based on systematic inflammatory response

**DOI:** 10.1093/jncics/pkad090

**Published:** 2023-11-06

**Authors:** Jia-Yi Qian, Lei-Lei Wu, Li-Yan Zhang, Kun Li, Zhi-Xin Li, Yong Zhao, Dong Xie

**Affiliations:** Department of Thoracic Surgery, Shanghai Pulmonary Hospital, School of Medicine, Tongji University, China; Department of Thoracic Surgery, Shanghai Pulmonary Hospital, School of Medicine, Tongji University, China; Shanghai Pulmonary Hospital, School of Medicine, Tongji University, China; Department of Respiratory Medicine, Renji Hospital, Shanghai Jiaotong University, School of Medicine, Shanghai, China; Department of Thoracic Surgery, Shanghai Pulmonary Hospital, School of Medicine, Tongji University, China; Department of Thoracic Surgery, Shanghai Pulmonary Hospital, School of Medicine, Tongji University, China; Department of Thoracic Surgery, Affiliated Hospital of Jiangnan University, Wuxi, China; Department of Thoracic Surgery, Shanghai Pulmonary Hospital, School of Medicine, Tongji University, China

## Abstract

**Background:**

This study aimed to construct an effective nomogram based on the clinical and laboratory characteristics to predict the prognosis of stage I lung adenocarcinoma with *EGFR* alteration.

**Methods:**

A retrospective study was performed of 913 eligible patients with *EGFR* alteration after surgery at Shanghai Pulmonary Hospital. The peripheral blood indicators were included in the nomogram. Calibration plots, concordance index, decision curve analysis, and X-tile software were used in this study. Recurrence-free survival (RFS) and overall survival were estimated by the Kaplan-Meier method and compared using the log-rank test.

**Results:**

Neutrophil to lymphocyte ratio and platelet to lymphocyte ratio were independent risk factors for RFS. The calibration curves for RFS probabilities showed good agreement between the nomogram prediction and actual observation. Furthermore, the nomogram, including neutrophil to lymphocyte ratio and platelet to lymphocyte ratio had a higher concordance index (0.732, 95% confidence interval = 0.706 to 0.758) than that without neutrophil to lymphocyte ratio or platelet to lymphocyte ratio (0.713, 95% confidence interval = 0.686 to 0.740), and decision curve analysis plots showed that the nomogram with neutrophil to lymphocyte ratio and platelet to lymphocyte ratio had better clinical practicability. Additionally, the patients were divided into 2 groups according to cutoff values of risk points, and statistically significant differences in RFS and overall survival were observed between the high-risk and low-risk groups (*P *<* *.001).

**Conclusions:**

High pretreatment levels of neutrophil to lymphocyte ratio and platelet to lymphocyte ratio were strongly associated with a worse prognosis in stage I *EGFR*-altered lung adenocarcinomas. Besides, the proposed nomogram with neutrophil to lymphocyte ratio and platelet to lymphocyte ratio presented a better prediction ability for the survival of those patients.

Lung cancer is 1 of the most common carcinomas in the world as well as the leading cause of cancer-related death worldwide (1,2). Even in patients with stage I non-small cell lung cancer (NSCLC), there is still a risk of tumor recurrence and metastasis after surgery. A multi-institutional study observed a 26% recurrence rate of stage I lung adenocarcinoma after complete surgical resection (3).

In the past, clinicians usually evaluated patient prognosis based on the TNM staging system, but the outcomes were heterogeneous. Thus, it is necessary to find a more accurate model to predict patient survival. A growing number of studies have shown that systematic inflammatory response is related to tumor proliferation, invasion, and metastasis and plays a considerable role in the process of tumor occurrence and growth (4,5). By measuring neutrophil to lymphocyte ratio, platelet to lymphocyte ratio, and Prognostic Nutritional Index, it was found that the presence of systematic inflammation was associated with poor prognosis of various malignant tumors, including NSCLC (6-9). The correlation between systematic inflammation and outcomes, however, remains controversial in early-stage NSCLC, especially in patients with *EGFR* alterations. Thus, this study aimed to explore the potential role of markers, including neutrophil to lymphocyte ratio, platelet to lymphocyte ratio, and Prognostic Nutritional Index in patients with stage I *EGFR*-altered lung adenocarcinoma. Moreover, the study aimed to construct a clinical nomogram for predicting long-term patient outcomes, which would help clinicians identify the high-risk population and promote individualized treatment and follow-up.

## Methods

### Patient selection

**Figure 1.**
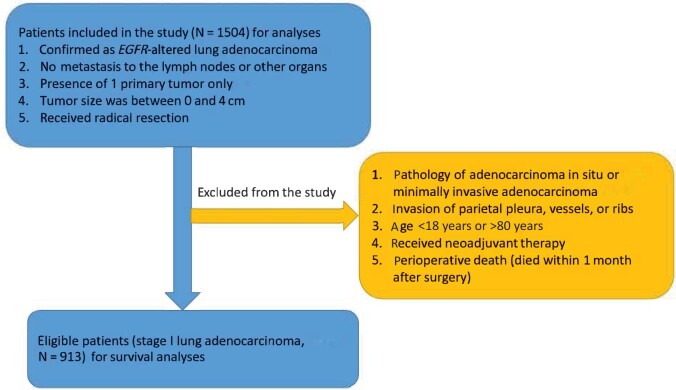
Study flowchart.

The study was conducted in accordance with the Declaration of Helsinki (as revised in 2013). Our study was composed of patients who underwent surgery for primary lung adenocarcinoma at Shanghai Pulmonary Hospital between 2015 and 2016. The patient selection criteria are presented in Figure 1. Information from 913 patients was collected, and patient TNM stage was identified according to the eighth edition TNM classification system. The clinical and laboratory data of patients considered several parts: age at surgery; gender; smoking history; surgery; tumor size; the predominant tumor pattern; types of *EGFR* alteration; spread through air spaces; visceral pleural invasion; lymphovascular invasion; adjuvant chemotherapy; and markers, including neutrophil to lymphocyte ratio, platelet to lymphocyte ratio, and Prognostic Nutritional Index.

### Follow-up and outcome

All patients were re-assessed after the surgery, and the relevant information was obtained through telephone calls or medical records. The follow-up duration ranged from 3.0 to 81.7 months, with an average of 58.8 months. Recurrence-free survival (RFS) was defined as the time from the date of surgery to the date of the first recurrence or last observation. Overall survival was calculated from the date of surgery to the date of death or the last day of follow-up.

### Preoperative variables

Peripheral blood test results were obtained preoperatively within 1 month before surgery, including the absolute neutrophil count, platelet count, absolute lymphocyte count, and albumin value. Complex parameters were calculated as follows: the neutrophil to lymphocyte ratio was calculated by division of absolute neutrophil and lymphocyte counts; the platelet to lymphocyte ratio was calculated as platelet count divided by absolute lymphocyte count; Prognostic Nutritional Index was calculated as 10 × albumin value (g/dL) + 0.005 × absolute lymphocyte count. To better evaluate the associations between these peripheral blood markers and patient outcomes, all 4 variables mentioned above were divided into high-level and low-level groups according to the cutoff values.

### Statistical analysis

The cutoff values of neutrophil to lymphocyte ratio, platelet to lymphocyte ratio, and Prognostic Nutritional Index for RFS were calculated using X-tile software (Camp/Rimm, Yale University). According to the calculated cutoff values, neutrophil to lymphocyte ratio, platelet to lymphocyte ratio, and Prognostic Nutritional Index were divided into low-level and high-level groups (Supplementary Figure 1, available online).

The proportions of categorical outcomes were assessed by Pearson χ^2^ test or Fisher exact test. The *t* test and Mann-Whitney *U* test were used to compare continuous variables in all cohorts. Univariate and multivariate Cox proportional hazards models were adopted to identify the independent prognostic predictors. Predictors (*P *<* *.05) in univariate analysis and known prognosis-affecting factors were brought into a multivariate analysis. Results of univariate and multivariate analyses were presented as hazard ratio (HR) and 95% confidence interval (CI), and 2-sided *P *<* *.05 was considered statistically significant.

Propensity score matching was applied to obtain 2 comparable patient populations by matching individual observations on their propensity scores. The ratio was 1:1, and the nearest-neighbor method was used.

Nomograms were constructed using R, version 4.1.1 (R Foundation for Statistical Computing, Vienna, Austria) based on the risk factors concluded from the multivariate analysis. The concordance index (C index) was measured by comparing predicted survival with the observed survival probability to better clarify the independent discrimination performance of the constructed nomogram: The larger the C index, the more accurate the prognostic stratification. The calibration was assessed by a calibration curve. The standard curve is a straight line passing through the origin of the coordinate axis, with a slope of 1. If the predicted calibration curve is closer to the standard curve, the better the predictive ability of the nomogram. Decision curve analysis was used to determine the clinical practicability of models. The cutoff values for total risk points were assessed using X-tile software, and patients were divided into high-risk and low-risk groups based on the cutoff values of risk scores. Patients’ RFS and overall survival were analyzed by using the Kaplan-Meier method, and the differences were compared by log-rank test. Statistical analyses were conducted using SPSS, version 23.0, software (IBM Inc, Armonk, NY), and all survival curves were constructed using R, version 4.1.1, software.

## Results

### Patient characteristics

Information from 913 patients was included in our study, and the baseline characteristics are summarized in Table 1; 335 (36.7%) patients were male, and 578 (63.3%) patients were female. The median age of the entire cohort was 61 years (interquartile range [IQR] = 55-67). Among enrolled patients, 821 (89.9%) patients underwent lobectomy, and only 92 (10.1%) patients underwent sublobectomy. Predominant patterns were as follows: lepidic, 328 (35.9%); acinar/papillary, 555 (60.8%); and micropapillary/solid, 30 (3.3%). The numbers of patients in the adjuvant chemotherapy and nonadjuvant chemotherapy groups were 237 (26.0%) and 676 (74.0%), respectively. According to the calculated cutoff values, there were 354 (38.8%) patients in the low-level (neutrophil to lymphocyte ratio ≤1.53) group and 559 (61.2%) patients in the high-level (neutrophil to lymphocyte ratio >1.53) group, 647 (70.9%) patients in the low-level (platelet to lymphocyte ratio ≤130.81) group and 266 (29.1%) patients in the high-level (platelet to lymphocyte ratio >130.81) group, and 652 (71.4%) patients in the low-level (Prognostic Nutritional Index ≤53.80) group and 261 (28.6%) patients in the high-level (Prognostic Nutritional Index >53.80) group.

**Table 1.** Baseline patient clinicopathological characteristics (N = 913)

Variable	Value
Gender, No. (%)	
Male	335 (36.7)
Female	578 (63.3)
Age at surgery, median (interquartile range), y	61 (55-67)
≤61, No. (%), y	469 (51.4)
≥62, No. (%), y	444 (48.6)
Smoking history, No. (%)	
No	791 (86.6)
Yes	122 (13.4)
Surgery, No. (%)	
Lobectomy	821 (89.9)
Sublobectomy	92 (10.1)
Predominant tumor pattern, No. (%)	
Lepidic	328 (35.9)
Acinar/papillary	555 (60.8)
Micropapillary/solid	30 (3.3)
Tumor size, No. (%)	
≤3 cm	817 (89.5)
3.1-4 cm	96 (10.5)
Visceral pleural invasion, No. (%)	
Absent	809 (88.6)
Present	104 (11.4)
Lymphovascular invasion, No. (%)	
Absent	907 (99.3)
Present	6 (0.7)
Spread through air spaces, No. (%)	
Absent	891 (97.6)
Present	22 (2.4)
*EGFR* alteration, No. (%)	
19-del	398 (43.6)
L858R	438 (48.0)
Others	77 (8.4)
Adjuvant chemotherapy, No. (%)	
No	676 (74.0)
Yes	237 (26.0)
Pathological stage, No. (%)	
IA	736 (80.6)
IB	177 (19.4)
Neutrophil to lymphocyte ratio, No. (%)	
≤1.53	354 (38.8)
>1.53	559 (61.2)
Platelet to lymphocyte ratio, No. (%)	
≤130.81	647 (70.9)
>130.81	266 (29.1)
Prognostic Nutritional Index, No. (%)	
≤53.80	652 (71.4)
>53.80	261 (28.6)

### Univariate and multivariate analyses

To identify the independent prognostic factors for patient outcomes after surgery, we analyzed patient RFS by using a Cox regression model. The multivariate analysis showed statistically significant prognostic values for 6 RFS factors: age at surgery 62 years or older (HR* *=* *1.606, 95% CI* *=* *1.063 to 2.426, *P *=* *.024), predominant pattern (micropapillary/solid, HR* *=* *5.805, 95% CI* *=* *2.331 to 14.459; acinar/papillary, HR* *=* *3.059, 95% CI* *=* *1.686 to 5.549; all *P *<* *.001), visceral plural invasion (HR* *=* *2.828, 95% CI* *=* *1.798 to 4.448, *P *<* *.001), lymphovascular invasion (HR* *=* *3.196, 95% CI* *=* *1.113 to 9.177, *P *=* *.031), neutrophil to lymphocyte ratio (HR* *=* *2.146, 95% CI* *=* *1.286 to 3.579, *P *=* *.003), and platelet to lymphocyte ratio (HR* *=* *1.591, 95% CI* *=* *1.031 to 2.456, *P *=* *.036) (Table 2). The effect of adjuvant chemotherapy and surgery on RFS among the overall population were not statistically significant.

**Table 2.** Univariate and multivariate analyses for recurrence-free survival

	Univariate analysis			Multivariate analysis		
Variable	Hazard ratio	95% confidence interval	*P*	Hazard ratio	95% confidence interval	*P*
Gender						
Male	1	—	—	—	—	—
Female	1.103	0.722 to 1.686	.650	—	—	—
Age at surgery, y						
≤61	1	—	—	1	—	—
≥62	1.503	1.003 to 2.252	.048	1.606	1.063 to 2.426	.024
Smoking history						
No	1	—	—	—	—	—
Yes	1.095	0.610 to 1.965	.761	—	—	—
Surgery						
Lobectomy	1	—	—	—	—	—
Sublobectomy	0.723	0.335 to 1.561	.409	—	—	—
Predominant pattern						
Lepidic	1	—	—	1	—	—
Acinar/papillary	3.474	1.928 to 6.260	<.001	3.059	1.686 to 5.549	<.001
Micropapillary/solid	7.409	3.070 to 17.879	<.001	5.805	2.331 to 14.459	<.001
Tumor size, cm						
≤3	1	—	—	—	—	—
3.1-4	1.663	0.972 to 2.844	.063	—	—	—
Visceral pleural invasion						
Absent	1	—	—	1	—	—
Present	3.448	2.230 to 5.332	<.001	2.828	1.798 to 4.448	<.001
Lymphovascular invasion						
Absent	1	—	—	1	—	—
Present	7.114	2.611 to 19.387	<.001	3.196	1.113 to 9.177	.031
Spread through air spaces						
Absent	1	—	—	—	—	—
Present	2.065	0.758 to 5.623	.156	—	—	—
*EGFR* alteration						
19-del	1	—	—	—	—	—
L858R	0.885	0.582 to 1.348	.570	—	—	—
Others	1.098	0.536 to 2.250	.798	—	—	—
Adjuvant chemotherapy						
No	1	—	—	—	—	—
Yes	1.644	1.085 to 2.491	.019	—	—	—
Neutrophil to lymphocyte ratio						
≤1.53	1	—	—	1	—	—
>1.53	2.322	1.431 to 3.768	.001	2.146	1.286 to 3.579	.003
Platelet to lymphocyte ratio						
≤130.81	1	—	—	1	—	—
>130.81	1.691	1.123 to 2.547	.012	1.591	1.031 to 2.456	.036
Prognostic Nutritional Index						
≤53.80	1	—	—	—	—	—
>53.80	0.726	0.451 to 1.169	.188	—	—	—

### Prognostic significance of neutrophil to lymphocyte ration and platelet to lymphocyte ratio

**Figure 2.**
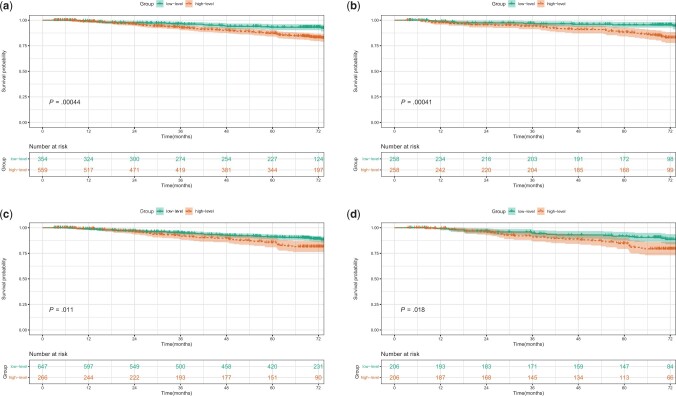
Kaplan-Meier survival curves for patients between the low-level (≤1.53) and high-level (>1.53) neutrophil to lymphocyte ratio groups before propensity score matching (**A**) and after propensity score matching (**B**) and Kaplan-Meier survival curves for patients between the low-level (≤130.81) and high-level (>130.81) platelet to lymphocyte ratio groups before propensity score matching (**C**) and after propensity score matching (**D**).

To further analyze the role of neutrophil to lymphocyte ratio and platelet to lymphocyte ratio in the patient prognosis after surgery, we excluded the interference of other factors by propensity score matching. Before propensity score matching, patients in the high-level neutrophil to lymphocyte ratio group were more likely to be male (*P *<* *.001) and smokers (*P *=* *.04), with a larger tumors (*P *=* *.001), and a higher neutrophil to lymphocyte ratio (*P *<* *.001) and lower platelet to lymphocyte ratio (*P *<* *.001). After matching, 258 pairs were included in the analysis, and there were no statistically significant differences between the 2 cohorts in terms of characteristics (Supplementary Table 1, available online). There were also statistically significant differences in age at surgery (*P *=* *.045), smoking history (*P *=* *.024), neutrophil to lymphocyte ratio (*P *<* *.001), and Prognostic Nutritional Index (*P *<* *.001) between the high-level and low-level platelet to lymphocyte ratio groups before the match. After propensity score matching, the differences among 206 pairs were balanced (Supplementary Table 2, available online). We found that patients with high neutrophil to lymphocyte and platelet to lymphocyte ratios were more likely to have a worse prognosis than those with low ratios before and after propensity score matching (Figure 2, A-D).

### Construction and validation of the prognostic risk model

**Figure 3.**
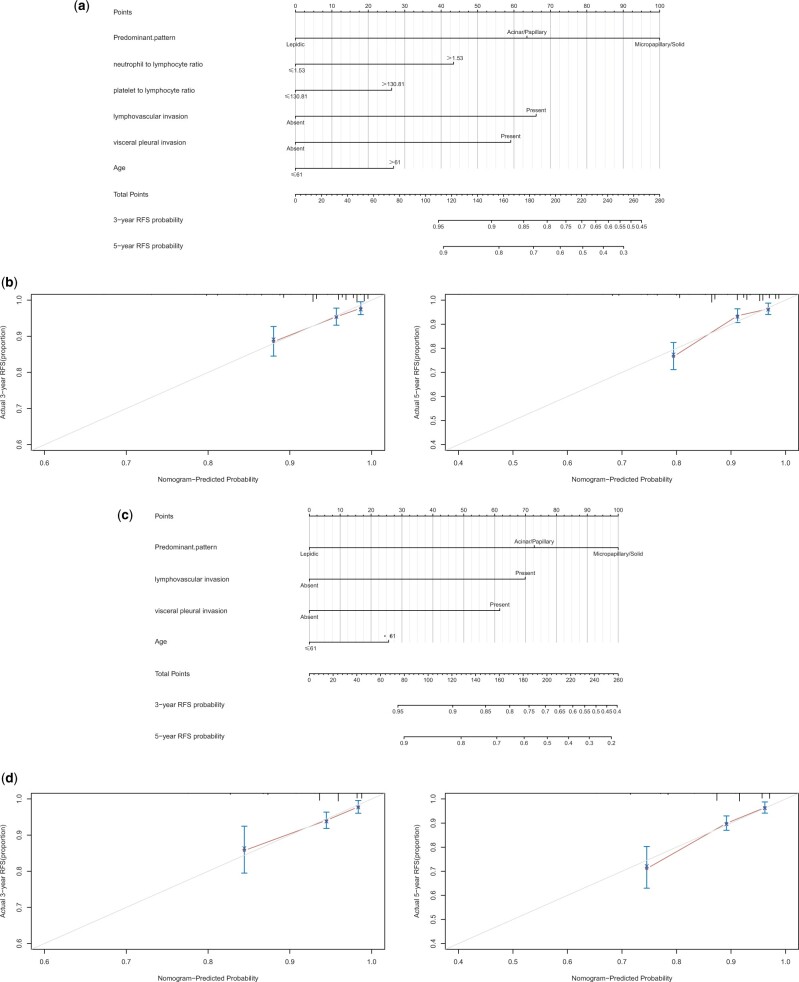
Nomogram with neutrophil to lymphocyte ratio and platelet to lymphocyte ratio for prediction of 3- and 5-year RFS (**A**) and calibration curves of the nomogram (**B**); nomogram without neutrophil to lymphocyte ratio or platelet to lymphocyte ratio for prediction of 3- and 5-year RFS (**C**) and calibration curves of the nomogram (**D**). RFS = recurrence-free survival.

To further study the impact of neutrophil to lymphocyte and platelet to lymphocyte ratios on the prognosis of patients with stage I *EGFR*-altered lung adenocarcinoma, we established nomograms with and without these peripheral blood markers. We first built a nomogram for RFS based on 6 independent prognostic factors: age at surgery, predominant pattern, lymphovascular invasion, visceral pleural invasion, neutrophil to lymphocyte ratio, and platelet to lymphocyte ratio (Figure 3, A). The calibration curves showed favorable agreement between the nomogram prediction and the actual observed outcomes of 3- and 5-year RFS (Figure 3, B). The C index of the nomogram with neutrophil to lymphocyte and platelet to lymphocyte ratios for predicting RFS was 0.732 (95% CI* *=* *0.706 to 0.758). We then constructed the nomogram for RFS without these ratios (Figure 3, C). The calibration curves also showed a favorable agreement between the nomogram prediction and the actual observed outcomes of 3- and 5-year RFS (Figure 3, D). The C index of the nomogram without the neutrophil to lymphocyte and platelet to lymphocyte ratios for predicting RFS was 0.713 (95% CI* *=* *0.686 to 0.740). In addition, the predictive ability of RFS was improved statistically significantly in the nomogram that included the neutrophil to lymphocyte and platelet to lymphocyte ratios than in the nomogram without them (*P *<* *.001).

### Clinical benefits of the nomograms

**Figure 4.**
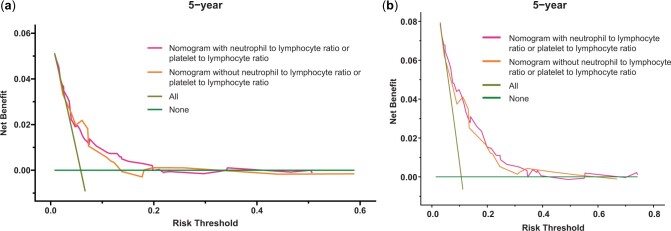
Decision curve analysis curves of the nomogram with the neutrophil to lymphocyte and platelet to lymphocyte ratios and the nomogram without these ratios for the 3-year (**A**) and 5-year (**B**) recurrence-free survival prediction.

The clinical benefits of the nomogram with neutrophil to lymphocyte and platelet to lymphocyte ratios and the nomogram without the ratios were compared. Decision curve analysis curves showed that the nomogram with the ratios could better predict the 3‐ and 5‐year RFS because it added more net benefits than the nomogram without the ratios for most threshold probabilities (Figure 4, A and B).

### Risk stratification based on the nomograms

**Figure 5.**
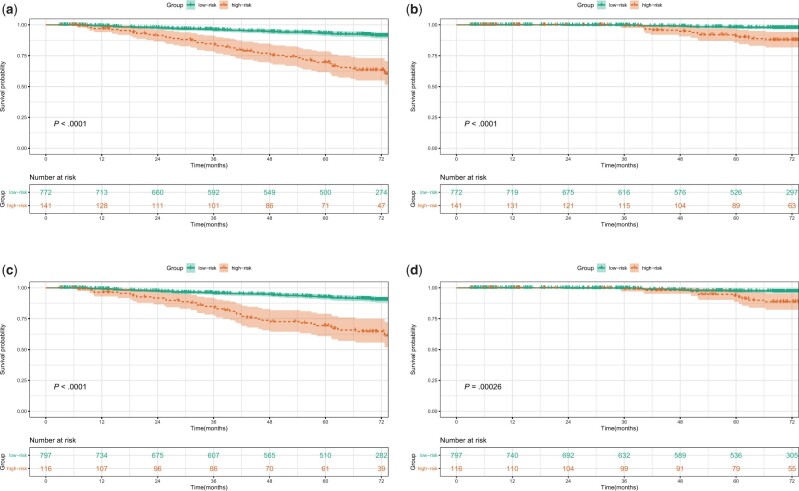
Kaplan-Meier survival curves for recurrence-free survival (**A**) and overall survival (**B**) between the high-risk and low-risk groups based on the nomogram with the neutrophil to lymphocyte and platelet to lymphocyte ratios. Kaplan-Meier survival curves for recurrence-free survival (**C**) and overall survival (**D**) between the high-risk and low-risk groups based on the nomogram without the neutrophil to lymphocyte or platelet to lymphocyte ratio.

We calculated the points of all patients and confirmed the optimal cutoff value using X-tile software. The cutoff values of sum points were 143.0 and 99.0, respectively, based on the nomogram with the neutrophil to lymphocyte and platelet to lymphocyte ratios and the nomogram without the ratios. All patients were classified into high-risk and low-risk groups according to the optimal cutoff values (Supplementary Figure 2, available online). In total, 141 patients were classified into the high-risk group based on the nomogram with the ratios, and 116 patients were classified into the high-risk group based on the nomogram without the ratios. The high-risk group had statistically significantly worse RFS (HR* *=* *5.249, 95% CI* *=* *3.512 to 7.846, *P *<* *.0001) and overall survival (HR* *=* *5.197, 95% CI* *=* *2.442 to 11.059, *P *<* *.0001) than the low-risk group based on the nomogram with the neutrophil to lymphocyte and platelet to lymphocyte ratios (Figure 5, A and B). The high-risk group had statistically significantly worse RFS (HR* *=* *4.680, 95% CI* *=* *3.102 to 7.062, *P *<* *.0001) and overall survival (HR* *=* *3.864, 95% CI* *=* *1.769 to 8.439, *P *=* *.00026) than the low-risk group based on the nomogram without neutrophil to lymphocyte and platelet to lymphocyte ratios (Figure 5, C and D).

## Discussion

The prognosis of early-stage NSCLC is highly heterogeneous and still has the possibility of recurrence leading to death after radical resection of the primary tumor. Previous studies have proposed that markers based on the test results in peripheral blood, including neutrophil to lymphocyte and platelet to lymphocyte ratios and Prognostic Nutritional Index, have yielded encouraging results in the prognostication of advanced NSCLC (10), but their role in patients with early-stage NSCLC is unclear, especially in those with *EGFR* alterations.

In the present study, we analyzed patient data from the Shanghai Pulmonary Hospital database to investigate whether these markers have predictive value in patient prognosis and whether they have an additive effect when combined with clinical risk factors. According to the results of multivariate analysis, 6 independent prognostic factors for RFS—age at surgery, visceral pleural invasion, lymphovascular invasion, predominant tumor pattern, neutrophil to lymphocyte ratio, and platelet to lymphocyte ratio—were identified. In contrast to Wang’s finding that the prognostic value of the neutrophil to lymphocyte ratio could be observed only in patients with wild-type tumors (11), our analysis showed that both high-level neutrophil to lymphocyte and platelet to lymphocyte ratios were strongly associated with worse prognosis in patients with stage I *EGFR*-altered lung adenocarcinoma. To further quantify and compare the impact of neutrophil to lymphocyte and platelet to lymphocyte ratios and other risk factors on patient prognosis, we constructed 2 nomograms: 1 with neutrophil to lymphocyte and platelet to lymphocyte ratios and another 1 without these ratios. We observed that the predominant tumor pattern, especially micropapillary/solid subtypes, had the greatest impact on patient outcomes in both models, which was consistent with the previous studies (12,13). Visceral pleural invasion and lymphovascular invasion, as well-known prognostic factors, were also strongly related to patient prognosis (14-17). The nomogram with neutrophil to lymphocyte and platelet to lymphocyte ratios had a higher C index (0.732) than the nomogram without these ratios (0.713), and decision curve analysis plots showed that the nomogram with neutrophil to lymphocyte and platelet to lymphocyte ratios had better clinical practicability. Furthermore, patients were categorized into high-risk and low-risk groups, with statistically significant differences in both RFS and overall survival based on the cutoff values of risk points. In addition, we found that the high-risk population defined by the nomogram with neutrophil to lymphocyte and platelet to lymphocyte ratios was larger in number and had a higher risk of recurrence and death. All this evidence suggested that high-level neutrophil to lymphocyte and platelet to lymphocyte ratios combined with clinical risk factors could result in a worse prognosis, and the nomogram with these ratios could better predict the outcomes of patients after surgery.

The Prognostic Nutritional Index is a score that represents patients’ immune nutritional status based on the lymphocyte count and serum albumin concentration. Several studies have proposed that the index is related to the prognosis of multiple malignancies, especially in patients with digestive system carcinomas (18,19). For patients with advanced lung cancer, the Prognostic Nutritional Index has also been proven to be a predictor of early progression (20), but its role in early-stage NSCLC remains controversial. Different from the results of Park’s study (9), we consider that patients with advanced lung cancer often have poor nutritional status because of cachexia, resulting in a low-level Prognostic Nutritional Index. This phenomenon is rarely seen, however, in patients with early-stage NSCLC.

A series of studies has revealed that nomograms present a more accurate prognostic prediction than the traditional staging system, but compared with the nomograms in our study, the previously established nomograms had fewer variables and lower C indies (21-23). Our study incorporated a large number of patients with *EGFR*-altered tumors and as many variables as possible to construct the model that could identify patients at high risk of recurrence and death, and the accuracy was proven.

It should be noted, however, that there were still some limitations in our study. First, all patient data were from a single-center database of Shanghai Pulmonary Hospital, with no external validation cohort, which resulted in a lack of representativeness. Second, for early-stage NSCLC, the follow-up duration of this study was relatively short. Therefore, multicenter and prospective observational studies are needed to collect more detailed variables and samples to validate the accuracy and practicability of the neutrophil to lymphocyte ratio and platelet to lymphocyte ratio in predicting the prognosis of patients with stage I *EGFR*-altered lung adenocarcinoma after surgery.

In summary, neutrophil to lymphocyte ratio and platelet to lymphocyte ratio were statistically significantly associated with poor prognosis among patients with stage I *EGFR*-altered lung adenocarcinoma after surgery, and the established nomogram with the neutrophil to lymphocyte and platelet to lymphocyte ratios could predict patient prognosis more accurately than the nomogram without these ratios.

## Supplementary Material

pkad090_Supplementary_Data

## Data Availability

The research data are available in the article itself and its Supplementary Material (available online). Other relevant clinical data will be shared upon any request.
