# Supplementary material for: Prognostic evaluation of stage I lung adenocarcinoma based on systematic inflammatory response
Source: JNCI Cancer Spectr. 2023 Nov 6;7(6):pkad090. doi: 10.1093/jncics/pkad090 (PMC10660118; doi:10.1093/jncics/pkad090)
Supplement: pkad090_Supplementary_Data [file pkad090_supplementary_data.pdf]

Supplementary Tables

Supplementary Table 1 Characteristics of patients in low-level and high-level groups of NLR before and after matching.

	Before PSM			After PSM		
**Variables**	**≤ 1.53 (N = 354)**	**> 1.53 (N = 559)**	***P* - value**	**≤ 1.53 (N = 258)**	**> 1.53 (N = 258)**	***P* - value**
Gender			＜ 0.001			0.636
Male	104 (29.4%)	231 (41.3%)		84 (32.6%)	79 (30.6%)	
Female	250 (70.6%)	328 (58.7%)		174 (67.4%)	179 (69.4%)	
Age at surgery, years (IQR)	61 (56-66)	61 (55-67)	0.525	61 (55-65)	61 (55-68)	0.283
Smoking history			0.040			0.588
No	317 (89.5%)	474 (84.8%)		225 (87.2%)	229 (88.8%)	
Yes	37 (10.5%)	85 (15.2%)		33 (12.8%)	29 (11.2%)	
Extent of surgery			0.229			0.770

Lobectomy	313 (88.4%)	508 (90.9%)		233 (90.3%)	231 (89.5%)	
Sub-lobectomy	41 (11.6%)	51 (9.1%)		25 (9.7%)	27 (10.5%)	
Predominant pattern			0.436			0.460
Lepidic	136 (38.4%)	192 (34.3%)		95 (36.8%)	95 (36.8%)	
Acinar/Papillary	206 (58.2%)	349 (62.5%)		152 (58.9%)	157 (60.9%)	
Micropapillary /Solid	12 (3.4%)	18 (3.2%)		11 (4.3%)	6 (2.3%)	
Tumor size, cm (IQR)	2.0 (1.5-2.5)	2.0 (1.5-2.5)	0.001	2.0 (1.5-2.5)	2.0 (1.5-2.5)	0.653
VPI			0.720			0.784
Absent	312 (88.1%)	497 (88.9%)		229 (88.8%)	227 (88.0%)	
Present	42 (11.9%)	62 (11.1%)		29 (11.2%)	31 (12.0%)	
LVI			0.487			0.616
Absent	353 (99.7%)	554 (99.1%)		257 (99.6%)	255 (98.8%)	
Present	1 (0.3%)	5 (0.9%)		1 (0.4%)	3 (1.2%)	

STAS			0.498			0.559
Absent	347 (98.0%)	544 (97.3%)		253 (98.1%)	251 (97.3%)	
Present	7 (2.0%)	15 (2.7%)		5 (1.9%)	7 (2.7%)	
EGFR mutation			0.866			0.379
19-del	157 (44.4%)	241 (43.1%)		124 (48.1%)	114 (44.2%)	
L858R	166 (46.8%)	272 (48.7%)		109 (42.2%)	124 (48.0%)	
Others	31 (8.8%)	46 (8.2%)		25 (9.7%)	20 (7.8%)	
ACT			0.168			0.838
No	271 (76.6%)	405 (72.5%)		193 (74.8%)	195 (75.6%)	
Yes	83 (23.4%)	154 (27.5%)		65 (25.2%)	63 (24.4%)	
PLR (IQR)	91.35 (71.88-110.26)	121.98 (95.61-150.87)	< 0.001	99.52 (84.11-116.96)	99.57 (84.62-118.99)	0.896
PNI (IQR)	52.75 (49.30-56.36)	50.35 (47.20-53.40)	< 0.001	51.75 (49.05-55.51)	52.22 (48.65-54.63)	0.814

Data are expressed as n (%) or median (IQR). IQR, interquartile range; ACT, adjuvant chemotherapy; VPI, visceral pleural invasion; LVI, lymphovascular

invasion; STAS, spread through air spaces. EGFR, epidermal growth factor receptor; NLR, neutrophil-to-lymphocyte ratio; PLR, platelet-to-lymphocyte ratio; PNI, prognostic nutritional index.

Supplementary Table 2 Characteristics of patients in low-level and high-level groups of PLR before and after matching.

	Before PSM			After PSM		
**Variables**	**≤ 130.81 (N = 647)**	**> 130.81 (N = 266)**	***P* - value**	**≤ 130.81 (N = 206)**	**> 130.81 (N = 206)**	***P* - value**
Gender			0.057			0.075
Male	250 (38.6%)	85 (32.0%)		47 (22.8%)	63 (30.6%)	
Female	397 (61.4%)	181 (68.0%)		159 (77.2%)	143 (69.4%)	
Age at surgery, years (IQR)	62 (56-67)	61 (54-66)	0.045	61 (53-65)	61 (54-66)	0.740
Smoking history			0.024			0.269
No	550 (85.0%)	241 (90.6%)		193 (93.7%)	187 (90.8%)	
Yes	97 (15.0%)	25 (9.4%)		13 (6.3%)	19 (9.2%)	
Extent of surgery			0.497			0.329
Lobectomy	579 (89.5%)	242 (91.0%)		182 (88.3%)	188 (91.3%)	
Sub-lobectomy	68 (10.5%)	24 (9.0%)		24 (11.7%)	18 (8.7%)	

Predominant pattern			0.452			0.314
Lepidic	225 (34.8%)	103 (38.7%)		84 (40.8%)	73 (35.4%)	
Acinar/Papillary	399 (61.7%)	156 (58.7%)		120 (58.2%)	128 (62.2%)	
Micropapillary /Solid	23 (3.6%)	7 (2.6%)		2 (1.0%)	5 (2.4%)	
Tumor size, cm (IQR)	2.0 (1.5-2.5)	2.0 (1.5-2.5)	0.434	2.0 (1.5-2.5)	2.0 (1.5-2.5)	0.547
VPI			0.766			0.767
Absent	572 (88.4%)	237 (89.1%)		181 (87.9%)	179 (86.9%)	
Present	75 (11.6%)	29 (10.9%)		25 (12.1%)	27 (13.1%)	
LVI			1.000			1.000
Absent	643 (99.4%)	264 (99.2%)		204 (99.0%)	204 (99.0%)	
Present	4 (0.6%)	2 (0.8%)		2 (1.0%)	2 (1.0%)	
STAS			0.105			0.615
Absent	628 (97.1%)	263 (98.9%)		205 (99.5%)	203 (98.5%)	

Present	19 (2.9%)	3 (1.1%)		1 (0.5%)	3 (1.5%)	
EGFR mutation			0.588			0.817
19-del	289 (44.7%)	109 (41.0%)		88 (42.7%)	86 (41.7%)	
L858R	304 (47.0%)	134 (50.4%)		99 (48.1%)	104 (50.5%)	
Others	54 (8.3%)	23 (8.6%)		19 (9.2%)	16 (7.8%)	
ACT			0.411			0.827
No	484 (74.8%)	192 (72.2%)		146 (70.9%)	148 (71.8%)	
Yes	163 (25.2%)	74 (27.8%)		60 (29.1%)	58 (28.2%)	
NLR (IQR)	1.55 (1.21-2.01)	2.25 (1.75-2.89)	＜ 0.001	1.90 (1.41-2.53)	2.03 (1.65-2.53)	0.051
PNI (IQR)	52.40 (49.10-55.10)	48.62 (46.40-51.76)	＜ 0.001	49.55 (46.71-52.40)	49.50 (47.19-52.01)	0.988

Data are expressed as n (%) or median (IQR). IQR, interquartile range; ACT, adjuvant chemotherapy; VPI, visceral pleural invasion; LVI, lymphovascular invasion; STAS, spread through air spaces. EGFR, epidermal growth factor receptor; NLR, neutrophil-to-lymphocyte ratio; PLR, platelet-to-lymphocyte ratio; PNI, prognostic nutritional index.

Supplementary Figures

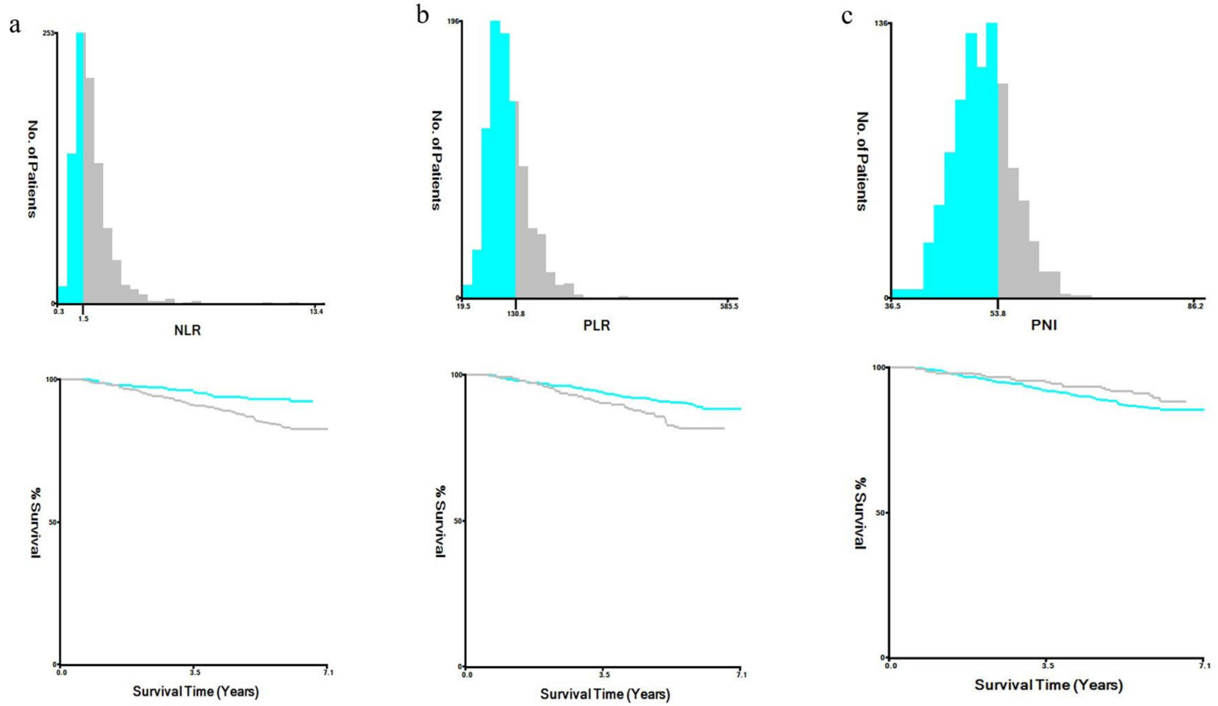

Supplementary Figure 1. The cut-off value for RFS of (a) NLR (≤ 1.53 was low-level group, > 1.53 was high-level group), (b) PLR (≤ 130.81 was low-level group, > 130.81 was high-level group), and (c) PNI (≤ 53.80 was low-level group, > 53.80 was high-level group) counted by X-tile. NLR: neutrophil-to-lymphocyte ratio; PLR: platelet-to-lymphocyte ratio; PNI: prognostic nutritional index.

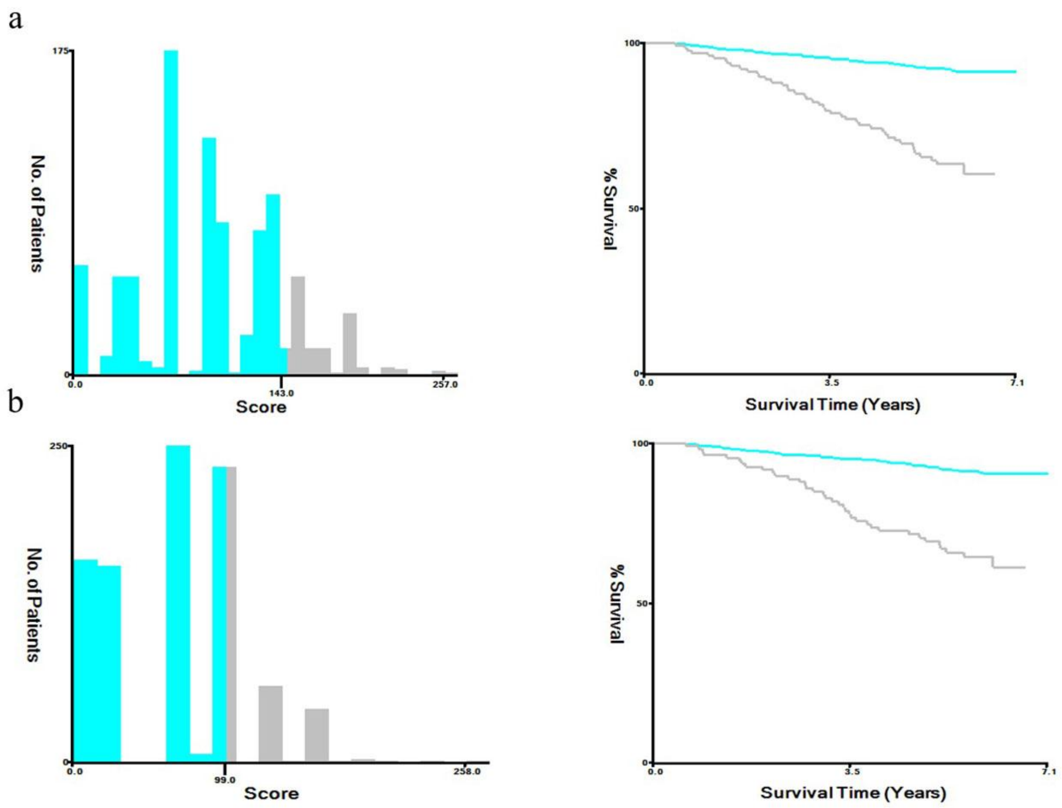

Supplementary Figure 2. The cut-off value of risk points counted by X-tile based on the nomogram with NLR and PLR (a) (points ≤ 143.0 was low-risk group, > 143.0 was high-risk group); and the cut-off value of risk points counted by X-tile based on the nomogram without NLR or PLR (b) (points ≤ 99.0 was low-risk group, > 99.0 was high-risk group). NLR: neutrophil-to-lymphocyte ratio; PLR: platelet-to-lymphocyte ratio.
